# Development of Processes for Recombinant L-Asparaginase II Production by *Escherichia coli* Bl21 (De3): From Shaker to Bioreactors

**DOI:** 10.3390/pharmaceutics13010014

**Published:** 2020-12-24

**Authors:** Thaís Barros, Larissa Brumano, Marcela Freitas, Adalberto Pessoa, Nádia Parachin, Pérola O. Magalhães

**Affiliations:** 1 Department of Pharmacy, Health Sciences School, University of Brasília, Brasília 70910-900, Brazil; thaisousabarros@hotmail.com (T.B.); marcelafreitas@aluno.unb.br (M.F.); 2 Department of Biochemical and Pharmaceutical Technology, University of São Paulo, São Paulo 05508-000, Brazil; larissabrumano@usp.br (L.B.); pessoajr@usp.br (A.P.J.); 3 Department of Cell Biology, Institute of Biology, University of Brasília, Brasília 70910-900, Brazil; nadiasp@unb.br

**Keywords:** L-asparaginase, *Escherichia coli*, IPTG, lactose, shaker, bioreactor

## Abstract

Since 1961, L-asparaginase has been used to treat patients with acute lymphocytic leukemia. It rapidly depletes the plasma asparagine and deprives the blood cells of this circulating amino acid, essential for the metabolic cycles of cells. In the search for viable alternatives to produce L-asparaginase, this work aimed to produce this enzyme from *Escherichia coli* in a shaker and in a 3 L bioreactor. Three culture media were tested: defined, semi-defined and complex medium. L-asparaginase activity was quantified using the β-hydroxamate aspartic acid method. The defined medium provided the highest L-asparaginase activity. In induction studies, two inducers, lactose and its analog IPTG, were compared. Lactose was chosen as an inducer for the experiments conducted in the bioreactor due to its natural source, lower cost and lower toxicity. Batch and fed-batch cultures were carried out to reach high cell density and then start the induction. Batch cultivation provided a final cell concentration of 11 g L^−1^ and fed-batch cultivation produced 69.90 g L^−1^ of cells, which produced a volumetric activity of 43,954.79 U L^−1^ after lactose induction. L-asparaginase was produced in a shaker and scaled up to a bioreactor, increasing 23-fold the cell concentration and thus, the enzyme productivity.

## 1. Introduction

Acute Lymphocytic Leukemia (ALL) is a neoplasia with the highest incidence in children, representing 76% of leukemia cases in patients under 15 years of age [1]. This neoplasm is characterized by the uncontrolled proliferation of immature lymphocytes (blasts), preventing the bone marrow to function normally [2]. Since 1961, the enzyme L-asparaginase (L-ASNase) combined with danorubicin, vincristine and prednisolone is used in the treatment of ALL, with a survival rate of 80% in pediatric patients [3].

L-ASNase (L-asparagine aminohydrolase, EC 3.5.1.1) catalyzes the hydrolysis of the amino acid asparagine into aspartic acid and ammonia [4]. This enzyme has been widely used in the food and pharmaceutical industries. In the food industry, L-ASNase is used to reduce the formation of acrylamide that is produced when foods rich in starch are cooked or fried through the Maillard reaction [5,6].

In the pharmaceutical field, L-ASNase is administered intravenously or intramuscularly, aiming at the rapid depletion of plasma asparagine (ASN), followed by the efflux of intracellular ASN depriving the cells of this circulating amino acid [7,8]. ASN is used in the metabolic cycles of the cell, and in its absence, the cell goes into apoptosis. However, unlike normal cells, cancer cells do not have or have low expression of the enzyme asparagine synthetase, being dependent on plasma ASN. Therefore, leukemic cells die of ASN starvation after depletion in plasma due to L-ASNase [7]. However, therapy with L-ASNase can cause significant effects such as anaphylaxis, coagulation abnormality, thrombosis, liver dysfunction, pancreatitis, hyperglycemia and cerebral dysfunction. These effects are due to the production of anti-asparaginase antibodies or by the glutaminase activity intrinsic to some L-ASNases [9].

In clinical practice, L-ASNase from two prokaryotic microorganisms are used, *Escherichia coli* and *Dickeya dadantii* (previously named as *Erwinia chrysanthemi*). *E. coli* L-ASNase formulations are used as line first treatment and *E. chrysanthemi* L-ASNase is used as second line treatment [8,10,11].

In the process of developing a product’s production, it is necessary to find suitable conditions that allow economically viable productivity and protein yield [12,13]. A combination of the production of recombinant proteins and large-scale processes has enabled the production of enzymes in greater quantities than found from their natural sources [14]. There is a wide variety of expression systems for the production of proteins, among them are cell cultures of bacteria, yeasts, fungi, mammals, plants, insects, transgenic and in vitro animals [15]. The *E. coli* is one of the most used hosts for the production of non-glycosylated heterologous because it presents advantages such as well-elucidated genetics and physiology, rapid growth, low cost of culture medium and allows high levels of protein expression enabling production and higher production scales [16,17]. For these advantages, *E. coli* was the expression system chosen for this work.

Aiming at the scarcity of studies on the production of recombinant L-ASNase in bioreactors, this work sought the production of this enzyme in a shaker and in high cell density in bioreactors up to 3 L, in order to define a detailed process for the production of L-ASNase in *E. coli* BL 21 (DE3).

## 2. Materials and Methods

### 2.1. Reagents

The reagents used were Ampicillin, Al_2_(SO_4_)_3_·16H_2_O, thiamine·HCl, ZnSO_4_·7H_2_O, IPTG, Tris, hydroxylamine hydrochloride, Asparagine, FeCl_3_, TCA and L-aspartic acid β-hydroxamate were Sigma-Aldrich™ (Steinheim, NW, Germany). The reagents KH_2_PO_4_, citric acid, MgSO_4_·7H_2_O, K_2_HPO_4_, ZnCl_2_, CaCl_2_·2H_2_O, CuSO_4_·5H_2_O, FeSO_4_·7H_2_O, NH_4_OH, HCl and lactose were Vetec Quimica Fina™ (Duque de Caxias, RJ, Brazil). The reagents glucose, (NH_4_)_2_HPO_4_, MnCl_2_·4H_2_O, Zn (CH_3_COO)_2_·H_2_O, CoCl_2_·6H_2_O, CuCl_2_·2H_2_O, EDTA·Na_2_, MnSO_4_·H_2_O, NiSO_4_·6H_2_O, Acetate and H_2_SO_4_ were Dinâmica Quimica™ (Indaíatuba, SP, Brazil). The reagents tryptone was Acumedia™, yeast extract was Kasvi™ (São José dos Pinhais, PR, Brazil), sodium chloride was Cromoline™ (Diadema, SP, Brazil), Glycerol was PlusOne™, Iron (III) Citrate was Êxodo científica™ (Sumaré, SP, Brazil), H_3_BO_3_ was Quimex™ (Uberaba, MG, Brazil), Na_2_MoO_4_·2H_2_O was Merck™ (Darmstadt, Germany) and NaH_2_PO_4_·H_2_O was labsynth™ (Diadema, SP, Brazil).

### 2.2. Microorganism

The experiments were performed using *E. coli* BL 21 (DE3) strain as host, kindly provided by Dr. Gisele Monteiro from the Faculty of Pharmaceutical Sciences of University of São Paulo. The expression vector used was pET-15b, which contains a codon gene optimized for expression of *E. coli* L-ASNase (GenBank KY305877), an export signal sequence to the periplasmic space and ampicillin resistance gene as a selective marker.

The standard stock was prepared by inoculating isolate colonies into a 250 mL Erlenmeyer flask with 50 mL of Luria Bertani (LB) medium (10.00 g L^−1^ of tryptone; 5.00 g L^−1^ of yeast extract; 5.00 g L^−1^ of sodium chloride and 0.10 g L^−1^ of ampicillin). This was incubated in an orbital shaker (New Brunswick series Innova 44 incubator, Eppendorf AG, Germany) at 37 °C and 180 rpm until the optical density at 600 nm (OD_600nm_) achieved values between 1.5 and 2.0. After centrifugation at 10,000× g for 10 min, the cells were suspended in 40 mL cryopreservation medium (85% LB medium and 15% glycerol). Aliquots were stored in cryotubes at −80 °C.

### 2.3. Culture Medium

For the production of L−ASNase, three culture media were evaluated: the defined medium [18], the semi-defined medium [19] and the complex medium [20]. The composition of the defined medium was 5.00 g L^−1^ of glucose; 13.30 g L^−1^ of KH_2_PO_4_; 4.00 g L^−1^ of (NH_4_)_2_HPO_4_; 1.70 g L^−1^ of citric acid; 1.20 g L^−1^ of MgSO_4_·7H_2_O; 0.06 g L^−1^ of Iron (III) Citrate; 1.50 × 10^−2^ g L^−1^ of MnCl_2_·4H_2_O; 0.80 × 10^−2^ g L^−1^ of Zn(CH_3_COO)_2_·H_2_O; 0.30 × 10^−2^ g L^−1^ of H_3_BO_3_; 0.25 × 10^−2^ g L^−1^ of Na_2_MoO_4_·2H_2_O; 0.25 × 10^−2^ g L^−1^ of CoCl_2_·6H_2_O; 0.15 × 10^−2^ g L^−1^ of CuCl_2_·2H_2_O; 0.84 × 10^−2^ g L^−1^ of EDTA·Na_2_; 0.45 × 10^−2^ g L^−1^ of thiamine.HCl. The composition of the semi-defined medium was 5.00 g L^−1^ of glucose; 1.00 g L^−1^ yeast extract; 13.00 g L^−1^ of KH_2_PO_4_; 10.00 g L^−1^ of K_2_HPO_4_; 3.00 g L^−1^ of (NH_4_)_2_HPO_4_; 4.60 g L^−1^ of NaH_2_PO_4_·H_2_O; 2.00 g L^−1^ of MgSO_4_·7H_2_O; 8.10 × 10^−2^ g L^−1^ of FeCl_3_·H_2_O; 0.39 × 10^−2^ g L^−1^ of ZnCl_2_; 0.60 × 10^−2^ g L^−1^ of CoCl_2_·6H_2_O; 7.38 × 10^−2^ g L^−1^ of CaCl_2_·2H_2_O; 3.81 × 10^−3^ g L^−1^ of CuCl_2_·2H_2_O; 0.15 × 10^−2^ g L^−1^ of H_3_BO_3_; 0.48 × 10^−2^ g L^−1^ of Al_2_(SO_4_)_3_·16H_2_O; 2.04 × 10^−2^ g L^−1^ of MnSO_4_·H_2_O; 0.60 × 10^−2^ g L^−1^ of Na_2_MoO_4_·2H_2_O. And the composition of the complex medium was 5.00 g L^−1^ of glucose; 5.00 g L^−1^ yeast extract; 10.00 g L^−1^ of Tryptone; 10.00 g L^−1^ of NaCl; 8.34 g L^−1^ of KH_2_PO_4_; 6.74 g L^−1^ of K_2_HPO_4_; 0.50 g L^−1^ of MgSO_4_·7H_2_O; 0.05 g L^−1^ of CaCl_2_·2H_2_O; 0.01 × 10^−2^ g L^−1^ of H_3_BO_3_; 0.01 × 10^−2^ g L^−1^ of CoCl_2_·6H_2_O; 2.50 × 10^−2^ g L^−1^ of ZnSO_4_·7H_2_O; 0.4 × 10^−2^ g L^−1^ of MnCl_2_·4H_2_O; 0.01 × 10^−2^ g L^−1^ of Na_2_MoO_4_·2H_2_O; 0.18 × 10^−2^ g L^−1^ of CuSO_4_·5H_2_O; 0.02 g L^−1^ of FeSO_4_·7H_2_O; 0.01 × 10^−2^ g L^−1^ of NiSO_4_·6H_2_O. All media were supplemented with 0.10 g L^−1^ of ampicillin.

### 2.4. Production of L-ASNase in Orbital Shaker

The inoculum was prepared with 0.25 mL of the cell stock in a 250 mL Erlenmeyer flask containing 25 mL of the defined, semi-defined or complex medium supplemented with 0.10 g L^−1^ of ampicillin and incubated in the orbital shaker at 37 °C and 180 rpm for up to 16 h.

Cultures were performed in an orbital shaker in 500 mL Erlenmeyer flasks containing 50 mL of the defined, semi-defined or complex medium with an initial OD_600nm_ of 0.1. Aliquots of 0.50 mL were taken during the culture and the total volume removed was less than 10% of the initial volume. The cultures were performed in triplicates.

Two inducers were used in this work, lactose and the isopropyl analog β-D-1-thiogalactopyranoside (IPTG). The lactose stock solution was prepared at a concentration of 200 g L^−1^ in distilled water and sterilized by moist heat. The IPTG stock solution was prepared at a concentration of 1.00 mol L^−1^ in distilled water and sterilized by 0.22 µm membrane filtration.

To determine the best concentration of each inductor, three concentrations were chosen based on previous studies. For IPTG, concentration often ranges from 0.10 mmol L^−1^ to 1.00 mmol L^−1^ [21,22,23,24] while lactose concentration varies from 10 g L^−1^ to 18 g L^−1^ [25,26,27]. Considering the reported concentrations, IPTG was evaluated at 0.10 mmol L^−1^, 0.45 mmol L^−1^ and 1.00 mmol L^−1^ and lactose was evaluated at 10 g L^−1^, 14 g L^−1^ and 18 g L^−1^.

### 2.5. Production of L-ASNase in Bioreactor

For cultures in the bioreactor, the New Brunswick BioFlo/CelliGen^TM^ 115 bioreactor (Eppendorf AG, Hamburg, Germany) was used. Vessels with capacities of 1.30 L and 3.00 L were used for batch and fed-batches, respectively. The glucose concentration in the culture medium was adjusted to 27.50 g L^−1^. At 37 °C, pH 6.8, it was not necessary to add acid, only base (NH_4_OH 3 M). Dissolved oxygen (DO) was kept at a minimum of 40%, by the agitation cascade of 200 to 900 rpm and 2 vvm of compressed air/oxygen were used during the process.

Feeding started after the peak of dissolved oxygen (DO-stat), observed when the initial glucose consumption was completed. The composition of the feeding solution was 600.00 g L^−1^ of glucose, 20.00 g L^−1^ of MgSO_4_·7H_2_O, 8.13 × 10^−1^ g L^−1^ of EDTA, 0.04 g L^−1^ of Iron (III) citrate, 0.04 × 10^−1^ g L^−1^ of CoCl_2_·2H_2_O, 2.35 × 10^−2^ g L^−1^ of MnCl_2_·4H_2_O, 0.23 × 10^−2^ g L^−1^ of CuCl_2_·2H_2_O, 0.47 × 10^−2^ g L^−1^ of H_3_BO_3_, 0.04 × 10^−1^ g L^−1^ of Na_2_MoO_4_·2H_2_O and 0.16 × 10^−1^ g L^−1^ of Zn(CH_3_COO)_2_·H_2_O. The exponential feeding strategy was chosen to feed the cultivation, therefore it was necessary to obey the mathematical model derived from the mass balance assuming constant cellular yield [14]. Equation (1) is described below:

(1) $$F=\left(\frac{\mu }{Y_{x/s}}+m\right)X\left(t_{0}\right)V\left(t_{0}\right)exp\left[\mu \left(set\right)\;\left(t-t_{0}\right)\right]$$

where, *F* is the feed flow rate (L h^−1^); *µ* is the actual specific growth rate (h^−1^) and *µ(set)* the desired specific growth rate (h^−1^); *Y_x/s_* is the biomass/substrate coefficient (g g^−1^); *m* is the specific maintenance coefficient (g g^−1^ h^−1^); *X(t*_0_*)* is the concentration of biomass at the beginning of feeding (g L^−1^); *V* is the cultivation volume at the beginning of feeding (L) and *t-t*_0_ is the feeding time elapsed (h). The cultivations were performed in duplicates.

### 2.6. L-asparaginase Activity Assay

To quantify L-ASNase activity, the L-aspartyl-β-hydroxamate method (AHA) was employed, which measures the amount of L-aspartic acid β-hydroxamate released in the hydrolysis reaction catalyzed by the enzyme L-ASNase from the amino acid asparagine in the presence of hydroxylamine [28]. The assay was performed in 5 mL conical tubes. Test tubes were prepared by adding 1.50 mL of Tris-HCl buffer (50.00 mmol L^−1^, pH 8.6), 0.20 mL of asparagine (0.10 mol L^−1^), 0.20 mL of hydroxalamine (1.00 mol L^−1^, pH 7.0) and 0.05 × 10^−1^ g of the sample. The sample blanks were prepared by adding 1.90 mLTris-HCl buffer (50.00 mmol L^−1^, pH 8.6) to 0.05 × 10^−1^ g of the sample and the tubes were incubated at 37 °C for 30 min. The reaction was stopped with 0.50 mL of ferric chloride reagent (10% (*w*/*v*) FeCl_3_, 20% (*w*/*v*) TCA and 0.66 M HCl solution). The tubes were centrifuged at 10,000× g for 10 min and the absorbance of the supernatant was measured in a spectrophotometer at 500 nm. The experiments were performed in independent biological triplicates and the activity of L-ASNase was quantified in triplicate. A standard curve was made with L-aspartic acid β-hydroxamate (0.10, 0.25, 0.50, 0.75, 1.00, 1.50, 2.00 and 3.00 µmol) in Tris-HCl buffer (50.00 mmol L^−1^, pH 8.6) with 0.50 mL of ferric chloride reagent, in triplicate. The equation of the line generated was y = 0.2967x + 0.0058 with that of R^2^ = 0.9957. L-ASNase activity was expressed as U g^−1^ of wet cells in Equation (2).

(2) $$\frac{U}{g}L-ASNase=\left[\frac{\left(µ\mathrm{mol}\;L-\mathrm{Aspartic}\;\mathrm{acid}\;\beta -\mathrm{hydroxamate}\right)}{\left(\mathrm{sample}\;\mathrm{weight}\;\left(g\right)\right)\times \left(\mathrm{reaction}\;\mathrm{time}\;\left(\min\right)\right)}\right]$$

Glucose, acetate and lactose were quantified using a Prominence UFLC ((Shimadzu^TM^, Kyoto, Japan)) chromatograph equipped with SPD-20A (Shimadzu^TM^) and RID-10A (Shimadzu^TM^) detectors. A Shim-Pack SCR-101H (7.9 mm × 30 cm, Shimadzu^TM^) ion exclusion column was used. Samples were analyzed using 5.00 mmol L^−1^ H_2_SO_4_ as a mobile phase in isocratic elution with a sample injection volume of 20.00 µL, 0.60 mL/min flow rate and column temperature of 60 °C.

### 2.7. Determination of Cell Dry Weight

For determination of cell dry weight, 2.00 mL conical tubes were used. The tubes were washed with distilled water and dried at 70 °C for 24 h. After cooling to room temperature, they were weighed on an analytical scale. Aliquots (1.00 mL) of the cell culture (OD_600nm_ = 0.10, 0.20, 0.30, 0.50, 1.00, 1.20, 1.40) were transferred to the previously weighed tubes, in triplicate, centrifuged for 10 min at 10,000× g and the cell pellet was washed with 2.00 mL of distilled water. Finally, the tubes were placed to dry in an oven at 70 °C up to constant weight. After plotting the OD_600nm_ data versus cell dry weight, the equation of the line y = 1.7242x + 0.0745 with that of R^2^ = 0.9902 was obtained.

### 2.8. Statistical Analysis

Statistical analyses were performed using GraphPad Prism^TM^ (San Diego, CA, U.S.A.) software version 6.01. Data distribution was assessed for normal distribution. For the data that presented normal distribution, it was applied a parametric test of variance analysis (ANOVA)-Dunnet, and the data were represented by the mean and standard deviation. The significant difference was considered for the values of *p* ≤ 0.05.

## 3. Results and Discussion

### 3.1. Assessment of the Culture Medium

Three culture media were evaluated in shake flasks: the defined medium, the semi-defined medium and the complex medium. The common carbon source among the three media, glucose, was fixed at 5.00 g L^−1^. For each medium, a growth curve was constructed and the final dry weight (X), the specific growth rate (µ_x_) and biomass yield (Y_x/s_) were calculated. The medium that presented the highest cellular growth after 10 h of cultivation was the complex medium with 3.24 g L^−1^, followed by the defined medium with 1.80 g L^−1^ and finally the semi-defined medium with 1.48 g L^−1^ (Table 1).

The highest µ_x_ was achieved by the complex medium with 0.81 h^−1^ and the lowest µ_x_ was found in the defined medium (0.55 h^−1^). The induction of the expression was performed by adding 0.45 mmol L^−1^ of IPTG, after three hours from the beginning of the cultivation. The culture was stopped after 18 h of induction. The highest enzyme activity was observed in the defined medium, with 94.77 ± 10 U g_cell_^−1^, followed by the complex medium with 58.10 ± 5U g_cell_^−1^ and the semi-defined medium with the lowest L-ASNase activity of 39.00 ± 10 U g_cell_^−1^.

The µ_x_ was analyzed in the exponential phase of each medium. The complex medium presented the largest µ_x_. It is known that there is a linear relationship between µ_x_ and acetate production: the higher the µ_x_ the greater the acetate production [29,30]. The production of acetate must be controlled because in high concentrations acetate inhibits cell growth [31]. The complex medium presented the highest Y_x/s_. The higher cellular concentration of the complex medium can be explained due to the fact that in addition to glucose, the complex medium has two more carbon sources, yeast extract and tryptone, thus causing the greatest cell growth. For the calculation of the Y_x/s_, only glucose was considered as a carbon source. The presence of yeast extract and tryptone in the composition of the semi-defined and complex media brings the difficulty of reproduction as a disadvantage due to the significant variation of these compounds between batches and difficult quality control. The defined medium has been gaining ground in commercial fermentations due to its greater reproducibility and rapid scaling process [32]. Therefore, the defined medium was chosen for the continuity of the experiments.

### 3.2. Optimization of Induction Phase Using Shaker Flasks

For the induction study, two inducers were compared, lactose and its analog, IPTG. The optimal inducer concentration, the ideal time to start induction and the ideal time to finish cultivation were determined for each inducer.

**Figure 1:**
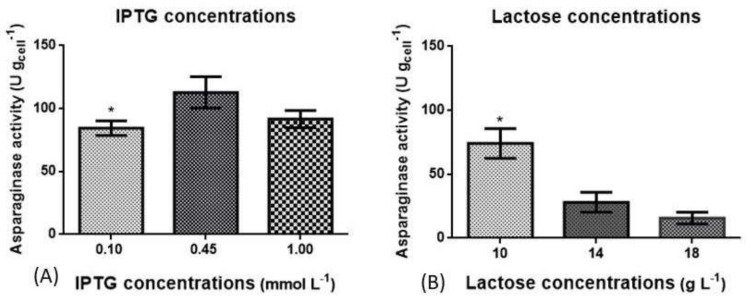
Evaluation of L-asparaginase activity (U g_cell_^−1^) of recombinant *E. coli* cultivated with different inductor concentrations. In (**A**) three concentrations of IPTG (0.10 mmol L^−1^, 0.45 mmol L^−1^ and 1.00 mmol L^−1^). were compared and expressed as In (**B**) three lactose concentrations (10 g L^−1^, 14 g L^−1^ and 18 g L^−1^) were compared. The results were expressed as mean and standard deviation. Cultivation was carried out in a defined medium, the initial OD_600nm_ was 0.1. After 5 h from the beginning of the cultivation, the inducer was added in different concentrations and after 24 h of induction the cultivation was stopped (*, *p* ≤ 0.05).

As it can be seen in Figure 1A, there was no significant difference in L-ASNase activity between IPTG concentrations of 0.45 mmol L^−1^ (112.81 ± 10 U g_cell_^−1^) and 1 mmol L^−1^ (91.69 ± 11 U g_cell_^−1^). As demonstrated in Figure 1B, the lactose concentration that best induced L-ASNase production with a significant difference was 10 g L^−1^, with mean L-ASNase activity of 74.15 ± 21 U g_cell_^−1^. The concentrations of 10 g L^−1^ and 0.45 mmol L^−1^ of lactose and IPTG, respectively, showed the highest L-ASNase activity values as demonstrated in Figure 1. Several studies used IPTG as an inducer for L-ASNase production (Table 2), with concentrations from 0.40 to 0.50 mmol L^−1^ being commonly used [33,34,35,36]. In previous studies, lactose was used at a concentration of 2 g L^−1^ reaching the specific activity 210 U mg^−1^ of the purified enzyme [37]. There is a difference in the final biomass concentration when comparing the cultivations induced with IPTG and lactose. The cultures induced with IPTG had an average final biomass of 1.92 g L^−1^ dry weight while the cultures induced with lactose had a final biomass of 2.67 g L^−1^ dry weight. This is due to fact that lactose serves as an inducer as well as a carbon source [25].

L-ASNase activity was evaluated after cultivation in the defined medium at four time points: at the beginning of the exponential phase, t = 3 h (OD_600nm_ between 0.4 to 0.6); middle of the exponential phase, t = 5 h (OD_600nm_ between 1.5 to 2.5); at the end of the exponential phase, t = 7 h (OD_600nm_ 3.8 to 4.0) and finally in the stationary phase, t = 9 h (OD_600nm_ 4.1 to 4.6). Cultivation was stopped after 24 h of induction.

**Figure 2:**
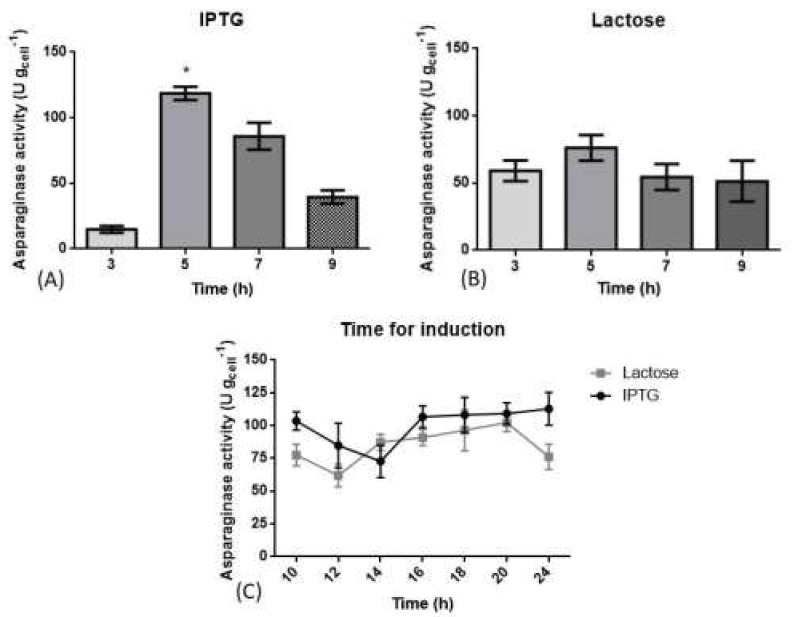
Study of L-ASNase production by recombinant *E. coli* induction with IPTG and lactose in a shaker. In (**A**), the induction was performed with IPTG varying the time of the beginning of the induction. In (**B**), the induction was performed with lactose, varying the induction start time. The cultures were induced in four moments, 3 h (OD_600nm_ between 0.4 and 0.6), 5 h (OD_600nm_ between 1.5 and 2.5), 7 h (OD_600nm_ between 3.8 and 4.0) and 9 h (OD_600nm_ between 4.1 and 4.6). (**C**) is a comparison between lactose and IPTG enzyme activities within 10–24 h after induction. The black line represents the IPTG-induced cultivation and the gray line, the lactose-induced cultivations. The results of L-asparaginase activities (U g_cell_^−1^). The results expressed as mean and standard deviation (*, *p* ≤ 0.05). The cultures were performed in a defined medium with the initial OD_600nm_ of 0.1 and concentration of inducers being 0.45 mmol L^−1^ of IPTG and 10 g L^−1^ of lactose.

As demonstrated in Figure 2A,B, the best time for L-ASNase production was in the middle of the exponential phase, after 5 h from the beginning of cultivation for both inducers. The culture induced with IPTG obtained L-ASNase activity of 115.99 ± 7U g_cell_^−1^ while in lactose-induced cultivation an L-ASNase activity of 76.21 ± 10 U g_cell_^−1^ was observed. Therefore, the exponential phase (5 h) was used as the time of induction in further experiments. Finally, L-ASNase activity was monitored between 10–24 h after induction. In Figure 2C, there was a plateau in L-ASNase activity of the IPTG-induced cultivation between 16–24 h with no statistical difference between them. In the lactose-induced cultivations, the best L-ASNase activity values were found between 18 h and 20 h, with no statistical difference between them.

The best time to start induction for both studied inducers was 5 h after the start of the cultivation, in the middle of the exponential phase, as described in Figure 2A,B. In previous studies, the time chosen for induction was when the OD_600nm_ was between 0.40 and 0.80 [38,47,51,52,53]. Since IPTG is toxic to cells, if added at the beginning of the cultivation there will be a loss in the final biomass concentration [27]. The interval of best L-ASNase activity was the same for both inducers used with no statistical difference between them. In the literature, is commonly found the induction ranged from 20 to 24 h after the start of cultivation [33,52,53].

Tian et al. (2011), compared the use of IPTG and lactose as inducers. The concentration of IPTG used was not described, and lactose with concentrations ranging from 2 to 50 g L^−1^ were evaluated for the production of keratinocyte growth factor-2. The highest production was achieved with 10 g L^−1^ of lactose, a result similar to that found in the present study [27]. Bashir et al. (2016), also reported a comparison between induction with IPTG and lactose, using IPTG concentrations of 1.00 mmol L^−1^, 1.50 mmol L^−1^ and 2.50 mmol L^−1^ and lactose concentrations from 2 to 16 g L^−1^. The authors concluded that 14 g L^−1^ of lactose showed the same level of induction as the concentration of 1.50 mmol L^−1^ IPTG for the production of recombinant consensus interferon [25]. The use of lactose as an inducer has advantages and disadvantages. As advantages it is possible to consider that it is of natural source, it has low cost, low toxicity and has a low induction speed, which is a benefit for the production of more soluble proteins [25,27]. As a disadvantage, lactose acts as both an inducer and as a carbon source during fermentation, which makes the process more difficult to control and requires higher amount a in the process when compared to IPTG [25]. Therefore, future studies that consider technical-economic analysis are needed to confirm which inducer (IPTG or lactose) is more advantageous for a scale-up process since they did not present statistically significant differences in each respective optimal concentration in the present work.

### 3.3. L Bioreactor Cultivation

Initially, batch cultivation was performed in order to obtain the kinetic parameters of recombinant *E. coli* strain.

**Figure 3:**
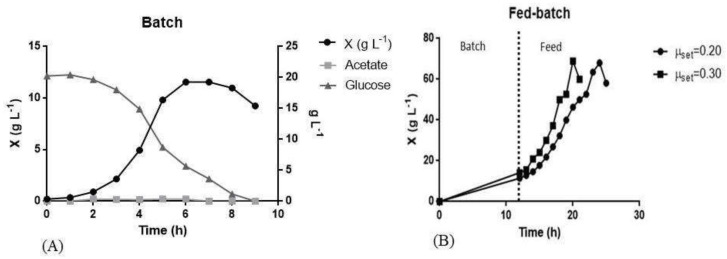
L-ASNase production of the recombinant *E. coli* cultivated in a bioreactor. (**A**) Batch cultivation. (**B**): Evaluation of µ_set_ 0.20 h^−1^ and 0.30 h^−1^ during exponential dry weight feeding (X). The experiments were carried out in biological duplicate and the figure shows a typical fermentation profile. The cultivations represented in (**A**) were carried out in a bioreactor with a capacity of 1.30 L and the initial OD_600nm_ of 0.4. The cultivations represented in (**B**) were carried out in a bioreactor with a capacity of 3 L and an initial OD_600nm_ of 0.01. Cultivations were stopped when biomass decayed.

As shown in Figure 3A, the maximum biomass obtained was 11 g L^−1^. The cultivation was interrupted when the OD_600nm_ started to decay. Glucose was completely consumed, and the residual acetate was 0.09 g L^−1^. The batch parameters are represented in Table 3.

In previous studies, where *E. coli* BL 21 was used for the production of peptide in the batch phase, the µ_x_ of 0.74 h^−1^ was calculated and the acetate concentration was below 0.50 g L^−1^ using complex medium [26]. In another work, the biomass reached 12 g L^−1^ during the batch phase, a similar result to that found in the present study (11 g L^−1^) using the same culture medium. The acetate production in the article was 1 g L^−1^, higher than that found in the present work of 0.09 g L^−1^ [18].

Taking the control of production into consideration, two values of µ_set_ were evaluated for the fed-batch: 0.20 h^−1^ and 0.30 h^−1^. In Figure 3B, the growth of biomass, glucose consumption and acetate production during feeding is presented. The batch phase is represented from 0 to 12 h. No samples were taken during this period since this phase was already well-described in the previous experiments of batch cultivations. After the DO peak, which represents the end of the initial glucose in the medium, feeding was started and samples were taken every hour. The maximum dry weight achieved was 69 g L^−1^, in both µ_set_, but at different times. Using µ_set_ of 0.30 h^−1^, the maximum biomass was reached with 8 h of feeding and using µ_set_ of 0.20 h^−1^, the maximum biomass was reached after 12 h of feeding. The residual acetate was 1.60 g L^−1^ and 2.56 at µ_set_ of 0.20 h^−1^ and 0.30 h^−1^, respectively, as described in Table 3. Oxygen was a limiting factor in both cultures. A mixture of 2 vvm compressed air and pure oxygen was added. The proportion of this mixture was adjusted according to the need for fermentation, reaching 100% oxygen.

Seeking to maximize volumetric productivity, high cell density cultivations were carried out, which has the advantage of reduced volumes in reactors, reduced residual water, easier cell separation and better product yield [18,70]. To achieve high cell density, continuous feeding of substrates is necessary for the production of intracellular and extracellular products [71]. Exponential rate feeding was developed in order to control the specific growth rate through the feed rate. An advantage of this type of feeding is to keep it below the critical value of acetate formation. In *E. coli* fermentations, it is desirable to maintain a low concentration of acetate because, in high concentrations, acetate will inhibit the growth and production of recombinant proteins, being described that the ideal µ_set_ would be between 0.10 h^−1^ and 0.30 h^−1^ so that acetate production during feeding would not inhibit cell growth [14]. As shown in Figure 3B, in both cultures µ_set_ of 0.20 h^−1^ or 0.30 h^−1^, dry mass reached 69 g L^−1^.

Few studies have reported the use of bioreactors for the production of L-ASNase by *E. coli*. Mihooliya and collaborators (2020) reported the achievement of OD_600nm_ 6 using a bioreactor with a capacity of 2 L and the cultivation was carried out in batch [34]. Roth and collaborators (2013) reached 30 g L^−1^ of dry weight using a bioreactor with a capacity of 2 L and the cultivation was carried out in fed-batch [58]. In a work published by Khushoo and collaborators (2005) the maximum biomass achieved was 53.30 g L^−1^ and the cultivation was carried out in fed-batch [72]. In the present work, a higher cell concentration of 69 g L^−1^ of dry cell weight (OD_600nm_ of 158) was achieved during the fed-batch.

**Figure 4:**
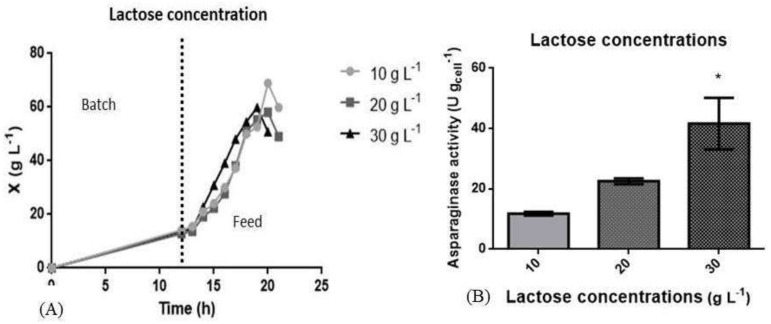
Study of induction of L-ASNase production by recombinant *E. coli* in a bioreactor with three concentrations of lactose (10 g L^−1^, 20 g L^−1^ and 30 g L^−1^). (**A**) shows the three growth curves in dry weight (g L^−1^) (X) and (**B**) shows the enzyme activity obtained by the different lactose concentrations used for induction. The experiments were carried out in biological duplicate and the figure shows a typical fermentation profile. The results of L-asparaginase activities (U g_cell_^−1^) were expressed as mean and standard deviation (*, *p* ≤ 0.05). Cultures were performed in a 3.00 L bioreactor with an initial OD_600nm_ of 0.01 in a defined medium. Cultures were induced when cell concentration reached 50 g L^−1^ and cultivation occurred when cell concentration declined.

The study of lactose concentration for induction was performed with three concentrations: 10 g L^−1^, 20 g L^−1^ and 30 g L^−1^. Induction was carried out when the cultivation reached 50 g L^−1^ dry weight and was interrupted when dry weight decay started. Figure 4A shows the comparison of the three growth curves that were induced with different concentrations of lactose, and Figure 4B shows the L-ASNase activity in U g_cell_^−1^. The culture that was induced with 10 g L^−1^ of lactose was the one with the highest cell concentration (69 g L^−1^) but presented the lowest L-ASNase activity (11.79 ± 0.53 U g_cell_^−1^). The culture that was induced with 20 g L^−1^ achieved a maximum cell concentration of 58 g L^−1^ and an increase in the value of L-ASNase activity (22.56 ± 0.93 U g_cell_^−1^). Finally, the culture that was induced with 30 g L^−1^ of lactose had a maximum cell concentration of 59 g L^−1^ and the highest L-ASNase activity (41.68 ± 10 U g_cell_^−1^).

Studies of lactose induction in a bioreactor were necessary due to the increase in cell concentration at the time of induction compared to culture in shaker flasks. The cultivation was induced with lactose in concentrations of 10, 20 and 30 g L^−1^. As shown in Figure 4A, less cell concentration was achieved in the cultures that used 20 and 30 g L^−1^ of lactose as an inducer, which can be attributed to the low solubility of lactose. Lactose has a solubility of 216 g L^−1^, thus requiring an increasing volume to reach the desired concentration in the culture. However, increased volume resulted in the dilution of the culture. Vélez et al. used pulses of 400 mL of lactose (200 g L^−1^) to induce the culture, to circumvent the low solubility of lactose for the production of Penicillin G acylase with maximum enzyme productivity of 7800 IU L^−1^h^−1^ [73].

**Figure 5:**
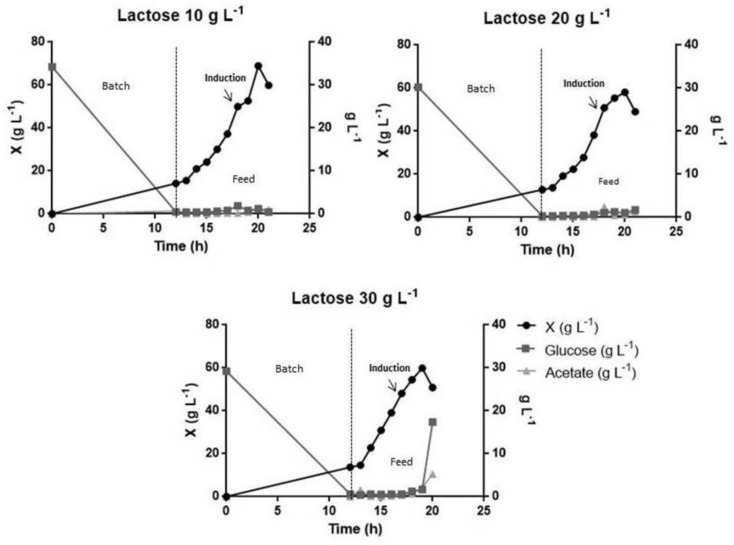
Representation of biomass growth, glucose consumption and acetate production in cultivations with different lactose concentrations. The ball represents the biomass (g L^−1^), the quadrate the glucose consumption (g L^−1^), the triangle the acetate production (g L^−1^) and the arrow represents the moment of lactose induction. Cultures were performed in a 3.00 L bioreactor with an initial OD_600nm_ of 0.01 in a defined medium. Cultures were induced when cell concentration reached 50 g L^−1^ and cultivation occurred when cell concentration declined, and the collected samples were analyzed in HPLC.

In Figure 4B, cultivation with lactose as an inducer at 30 g L^−1^ concentration provided the highest level of L-ASNase activity, the highest amount of unconsumed glucose and the highest production of acetate at the end of the cultivation in comparison to the other lactose concentrations used for induction, as shown in Figure 5 and in Table 3. This result could be explained by the hydrolysis of lactose in glucose and galactose, thus increasing the glucose concentration in the culture medium, thus excess glucose can explain the increase in acetate production. Furthermore, the reduction of the growth rate at this cultivation could be explained by the high concentration of acetate [31,74].

The culture induced with 30 g L^−1^ of lactose was the one that presented the highest production of acetate (5.25 g L^−1^), followed by 20 g L^−1^ (1.10 g L^−1^) and lastly, 10 g L^−1^ (0.96 g L^−1^) (Figure 5). The only culture that was interrupted without the complete consumption of glucose was the cultivation induced with 30 g L^−1^ of lactose.

The maximum lactose concentration used was 30 g L^−1^, not inducing the culture comparable to what was found in experiments performed in shaker flasks. Maximum L-ASNase activity achieved in shaker flasks was 99.00 U g_cell_^−1^ compared to 41.68 U g_cell_^−1^ achieved in a bioreactor. The specific activity of the crude extract was 1.08 U mg^−1^, with a maximum volumetric activity of 43,954.79 U L^−1^ in the bioreactor. The cell concentration reached in the bioreactor was 27-fold higher than in the shaker flasks.

Lactose induction optimization would require increasing the lactose concentration until it reaches the same level of induction as obtained when cultivation was carried out in shaker flasks. However, the increase in lactose concentration inside the bioreactor has a volume limitation, due to lactose low solubility. An increasing volume of the lactose solution would cause culture dilution, which may make the process unfeasible at an industrial level.

Kotzia and Labrou (2005) and Pourhossein and Korbekandi (2014) reported L-ASNase specific activity of 0.72 U mg^−1^ in the crude extract when working with the enzyme from *Erwinia carotovora* produced in *E. coli* [57,66]. A higher specific activity (1.08 U mg^−1^) was obtained in the present work. Chohan et al. (2018), obtained a volumetric activity of 20.13 U L^−1^ working with L-ASNase gene from *Pyrobaculum calidifontis* expressed in *E. coli*, lower than that found in the present study [42]. However, in a work published by Khushoo and collaborators (2005), the production of extracellular L-ASNase achieved the volumetric activity of 850,000 U L^−1^ in the extracellular medium, higher than that found in the present study [72].

## 4. Conclusions

This work carried out the production of the enzyme L-ASNase in recombinant *Escherichia coli* in a shaker and bioreactor using the defined medium and lactose as an inducer. The cell concentration was increased 27-fold when scaled up to a bioreactor, reaching about 70 g L^−1^ of dry weight and 43,954.79 U L^−1^ of volumetric activity. Despite the successful production of L-ASNase in a bioreactor, the increasing need for lactose concentration has become a problem during scaling, and new strategies are needed to optimize induction.

## Figures and Tables

**Table 1:** Final dry weight (X), specific growth rate (µ_x_) and biomass yield (Y_x/s_) and L-ASNase activity of the defined, semi-defined and complex media. For the quantification of L-ASNase activity, cultivation was carried out in each culture medium with an OD_600nm_ of 0.1, 3 h after the beginning of the cultivation 0.45 mmol L^−1^ of IPTG was added and after 18 h of induction the cultivation was stopped and the activity quantified by the AHA method.

Medium	X (g L^−1^)	µ_x_ (h^−1^)	Y_x/s_ (g g^−1^)	L-ASNase Activity (U g_cell_^−1^)
Defined	1.80	0.55	0.31	94.77 ± 10
Semi-defined	1.48	0.70	0.40	39.00 ± 10
Complex	3.24	0.81	0.63	58.10 ± 5

**Table 2:** Works published using *Escherichia coli* as an expression system for the production of L-asparaginase.

Gene Origin	Médium	L-ASNase Activity	Inductor	Reference
*Pseudomonas resinovorans*	Defined	38.88 UI mL^−1^ Δ	IPTG	[34]
*Anoxybacillus flavithermus*	LB	165 U mg^−1^ * Ο	IPTG	[38]
*Acinetobacter soli*	LB	400 U mg^−1^ * Ο	IPTG	[33]
*Thermococcus kodakarensis*	LB	767 U mg^−1^ * Ο	IPTG	[39]
*Cobetia amphilecti*	LB	778 U mg^−1^ * Ο	IPTG	[40]
*Lactobacillus casei*	LB	0.419 U mg^−1^ * Ο	IPTG	[41]
*Bacillus sp*	LB	-	IPTG	[35]
*Pyrobaculum calidifontis*	-	20.13 U L^−1^ Δ	IPTG	[42]
*Vibrio cholerae*	LB	2120 U mg^−1^ * Ο	IPTG	[43]
*Pseudomonas fluorescens*	LB	26 U mg^−1^ * Ο	IPTG	[44]
*Aspergillus terréus*	LB	42.46 U mg^−1^ * Ο	IPTG	[45]
*Halomonas elongata*	LB	1510 U mg^−1^ * Ο	IPTG	[46]
*Escherichia coli*	LB	-	IPTG	[36]
*Paenibaeillus barengoltzii*	LB	35.2 U mg^−1^ * Ο	IPTG	[47]
*Synechococcus elongatus*	LB	45 U mg^−1^ * Ο	IPTG	[48]
*Saccharomyces cerevisiae*	LB	110.1 UI mg^−1^ * Ο	IPTG	[49]
*Saccharomyces cerevisiae*	LB	196.2 U mg^−1^ * Ο	IPTG	[50]
*Erwinia carotovora*	LB	12.5 U mg^−1^ * Ο	IPTG	[51]
*Mesoflavibacter zeaxanthinifaciens*	LB	687.1 U mg^−1^ * Ο	IPTG	[52]
*Escherichia coli*	Defined	40.8 U mL^−1^ * Δ	IPTG	[53]
*Pseudomonas fluorescens*	LB	0.95 UI mg^−1^ * Ο	IPTG	[54]
*Rhodospirillum rubrum*	LB	210 U mg^−1^ *Ο	Lactose	[37]
*Escherichia coli*	LB	190 U mg^−1^ * Ο	IPTG	[55]
*Staphylococcus sp*	LB	113.06 U mg^−1^ * Ο	IPTG	[56]
*Erwinia carotovora*	LB	0.72 UI mg^−1^ * Δ	IPTG	[57]
*Erwinia carotovora*	LB	9.6 kcal mol^−1^ Ο	IPTG	[58]
*Yersinia pseudotuberculosis*	LB	-	Arabinose	[59]
*Yersinia Pseudotuberculosis*	LB	62.7 U mg^−1^ * Ο	Arabinose	[60]
*Withania somnifera L.*	LB	55 UI mg^−1^ * Ο	IPTG	[61]
*Yersinia pseudotuberculosis*	LB	62.7 UI mg^−1^ * Ο	Arabinose	[62]
*Flammulina velutipes*	LB	16 U mL^−1^ Δ	IPTG	[63]
*Glycine max*	-	-	IPTG	[64]
*Escherichia coli*	LB	130 U mL^−1^ Δ	-	[65]
*Erwinia carotovora*	LB	0.72 U mg^−1^ * Δ	IPTG	[66]
*Erwinia carotovora*	LB	630 UI mg^−1^ * Ο	IPTG	[67]
*Escherichia coli AS1.357*	LB	228 U mL^−1^ Δ	Heat	[68]
*Escherichia coli*	LB	91 U mg^−1^ * Ο	IPTG	[69]
*Escherichia coli*	Defined	1.08 U mg^−1^ * Δ	Lactose	This work

* = Specific activity; Δ = Crude extract; Ο = Purified enzyme; LB= Luria Bertani medium.

**Table 3:** Fermentation data of batch and fed-batch using recombinant *E. coli*. Specific growth rate (µ_x_), desired specific growth rate (µ_set_), Max dry weight (X_max_), biomass yield (Y_x/s_), glucose, acetate and residual lactose.

Fermentation		µ_x_ (h^−1^)	µ_set_ (h^−1^)	X _max_ (g L^−1^)	Y_x/s_ (g L^−1^)	Glucose (g L^−1^)	Acetate (g L^−1^)	Lactose (g L^−1^)
Batch		0.79	-	11.00	0.45	-	0.09	-
Batch fed	-	-	0.20	68.05	0.24	01.28	1.60	-
	-	-	0.30	68.98	0.25	00.86	2.56	-
Induction with lactose	10 g L^−1^	-	0.30	68.96	0.25	00.40	0.96	1.21
	20 g L^−1^	-	0.30	58.09	0.21	01.06	0.60	1.04
	30 g L^−1^	-	0.30	59.90	0.31	17.35	5.25	2.36
